# [^18^F]RO948 tau positron emission tomography in genetic and sporadic frontotemporal dementia syndromes

**DOI:** 10.1007/s00259-022-06065-4

**Published:** 2022-12-14

**Authors:** Alexander F. Santillo, Antoine Leuzy, Michael Honer, Maria Landqvist Waldö, Pontus Tideman, Luke Harper, Tomas Ohlsson, Svenja Moes, Lucia Giannini, Jonas Jögi, Colin Groot, Rik Ossenkoppele, Olof Strandberg, John van Swieten, Ruben Smith, Oskar Hansson

**Affiliations:** 1 Department of Clinical Sciences, Clinical Memory Research Unit, Faculty of Medicine, Lund University, Lund/Malmö, Sweden; 2 Memory Clinic, Skåne University Hospital, SE-20502 Malmö, Sweden; 3 Pharma Research and Early Development, Roche Innovation Center Basel, F. Hoffmann-La Roche, Basel, Switzerland; 4 Clinical Sciences Helsingborg, Department of Clinical Sciences Lund, Lund University, Lund, Sweden; 5 Radiation Physics, Skane University Hospital, Scania, Sweden; 6 Alzheimer Center, Department of Neurology, Erasmus Medical Center, Rotterdam, The Netherlands; 7 Clinical Physiology, Department of Clinical Sciences Lund, Lund University, Lund, Sweden; 8 Alzheimer Center Amsterdam, Department of Neurology, Amsterdam Neuroscience, Vrije Universiteit Amsterdam, Amsterdam UMC, Amsterdam, The Netherlands; 9 Department of Neurology, Skåne University Hospital, Lund, Sweden

**Keywords:** [^18^F]RO948, Tau, PET, Frontotemporal dementia, FTD, *C9orf72*, Progranulin, *MAPT*

## Abstract

**Purpose:**

To examine [^18^F]RO948 retention in FTD, sampling the underlying protein pathology heterogeneity.

**Methods:**

A total of 61 individuals with FTD (*n* = 35), matched cases of AD (*n* = 13) and Aβ-negative cognitively unimpaired individuals (*n* = 13) underwent [^18^F]RO948PET and MRI. FTD included 21 behavioral variant FTD (bvFTD) cases, 11 symptomatic *C9orf72* mutation carriers, one patient with non-genetic bvFTD-ALS, one individual with bvFTD due to a *GRN* mutation, and one due to a *MAPT* mutation (R406W). Tracer retention was examined using a region-of-interest and voxel-wise approaches. Two individuals (bvFTD due to *C9orf72*) underwent postmortem neuropathological examination. Tracer binding was additionally assessed in vitro using [^3^H]RO948 autoradiography in six separate cases.

**Results:**

[^18^F]RO948 retention across ROIs was clearly lower than in AD and comparable to that in Aβ-negative cognitively unimpaired individuals. Only minor loci of tracer retention were seen in bvFTD; these did not overlap with the observed cortical atrophy in the cases, the expected pattern of atrophy, nor the expected or verified protein pathology distribution. Autoradiography analyses showed no specific [^3^H]RO948 binding. The R406W *MAPT* mutation carriers were clear exceptions with AD-like retention levels and specific in-vitro binding.

**Conclusion:**

[^18^F]RO948 uptake is not significantly increased in the majority of FTD patients, with a clear exception being specific *MAPT* mutations.

**Supplementary Information:**

The online version contains supplementary material available at 10.1007/s00259-022-06065-4.

## Introduction

Positron emission tomography (PET) ligands binding to aggregates of the protein tau, the main pathological hallmark of Alzheimer’s disease (AD) alongside amyloid-β (Aβ), are powerful biomarkers that promise improved diagnostics, endpoints in treatment development studies and an improved understanding of AD pathophysiology [1]. Several tau ligands are now available [2], one of which [^18^F]flortaucipir (FTP) is approved by the US Food and Drug Administration (FDA) for AD diagnostics at the dementia stage [3]. The ultimate usefulness of tau tracers will depend not only on them capturing AD pathology but also their ability to separate AD pathology from non-AD neurodegenerative conditions (i.e., show specificity). Reports show that [^18^F]flortaucipir has uptake (either specific or off-target binding) in non-AD neurodegenerative conditions such as progressive supranuclear palsy (PSP), corticobasal syndrome (CBD), and frontotemporal dementia (FTD) [2], limiting its use as a differential diagnostic tool. A tau PET tracer with improved specificity would thus be beneficial.

From the perspective of molecular imaging, FTD is an elusive target since it is neuropathologically heterogenous. The majority of cases are characterized by the accumulation of either tau (either 3 or 4 repeat tau, as opposed to the combination of 3 and 4 repeat isoforms present in AD), TDP-43 (with its subtypes TDP types A, B, C, and D), or fused in sarcoma (FUS) protein [4]. Neuropathological examination is decisive to identify the specific protein pathology, but, still, clinical information may give valuable clues as to what neuropathology to expect. Genetic variants of FTD are very strongly associated with a particular protein pathology, with FTD due to mutations in the microtubule-associated protein tau (*MAPT*) gene, showing (as expected) tau pathology, whereas FTD due to mutations in the progranulin (*GRN*) gene and in chromosome 9 open reading frame 72 (*C9orf72*) shows TDP-43 pathology (of the types A and A or B, respectively) [4, 5]. FTD consists of several clinical syndromes; the most common is when behavioral symptoms dominate (behavioral variant of frontotemporal dementia; bvFTD) followed by two language-dominant syndromes, the semantic variant and the nonfluent/agrammatic variant of primary progressive aphasias (svPPA and nfvPPA) [4, 5]. The language variant svPPA is strongly (circa 90%) associated with TDP-43, particularly TDP-43 type C [4, 5], whereas nfvPPA is mostly due to tau pathology [6]. Ten to 15% of FTD cases have concomitant amyotrophic lateral sclerosis (ALS) [7], which also points strongly toward TDP-43 pathology, particularly type B [4, 5]. Cases with bvFTD without ALS (which is the most common FTD syndrome) are roughly evenly split between TDP-43 and tau pathology [8]. [^18^F]Flortaucipir, which is, by far, the most studied of the tau PET tracers, shows a varying binding to 4 repeat tauopathies [2]. In TDP-43 conditions [^18^F]flortaucipir shows varying tracer retention that is not explained by specific in vitro binding but, possibly, off-target binding [9–14]. The second-generation PET tracer [^18^F]RO948 has been shown in our previous study [15] to perform well in discriminating AD from cognitively unimpaired individuals. The purpose of this study is to examine binding of [^18^F]RO948 in FTD, particularly in cases (by genetic mutation or clinical syndrome) in which the underlying molecular pathology can be predicted ante mortem with high certainty. 

## Methods

### Participants

The 61participants are all from the prospective Swedish BioFINDER-2 study, (clinical trial no. NCT03174938, https://www.biofinder.se) performed at Lund University, Sweden, and included between 2017 and 2020. All individuals were recruited at a memory clinic and diagnosed by multidisciplinary assessment after clinical and neuropsychological examination, brain MRI, and lumbar puncture. For the present study, the participants were selected who fulfilled criteria for bvFTD according to the International Behavioral Variant FTD Consortium Criteria [16] (either probable or definite bvFTD) or had a genetic mutation and fulfilled criteria for PPA [17] or Petersen criteria for mild cognitive impairment (MCI) [18]. Screening for genetic mutation was not done consistently but on clinical grounds. CSF AD biomarkers or results of the RO948 PET were not used in the FTD diagnostic process. Twelve of the FTD cases (without knowledge of genetic status), and the case with *MAPT* mutation, have at ROI but not the voxel level been previously reported [15]. The patients with a diagnosis of AD dementia and the Aβ-negative cognitively unimpaired (CU) individuals were selected as to match genetic FTD cases in age and gender. The criteria for AD were fulfillment of DSM-5 criteria for dementia (major neurocognitive disorder) due to Alzheimer’s disease [19], a mini-mental state examination (MMSE) score of  ≥ 12 points, fluency in Swedish, and a positive Aβ status. Criteria for Aβ-negative CU were absence of cognitive symptoms as assessed by a memory clinic physician, a mini-mental state examination (MMSE) score of 26–30, not fulfilling criteria for MCI or any dementia according to DSM-5 and fluency in Swedish. Aβ status was determined using the CSF Aβ42/Aβ40 ratio with a cutoff of  < 0.089, as previously described [15].

### Ethics

All the participants gave written informed consent. Ethical approval was given by the Regional Ethical Committee in Lund, Sweden. Approval for PET imaging was additionally obtained from the Swedish Medicines and Products Agency and the local Radiation Safety Committee at Skåne University Hospital, Sweden.

### PET acquisition and processing

The participants were all scanned using a digital GE Discovery MI PET/CT machine (General Electric Medical Systems) after being injected with 365 ± 20 MBq of [^18^F]RO948, produced at Skane University Hospital. Acquisition time was 70–90 min [^18^F]RO948 post injection using the list-mode acquisition as described previously [15]. Low-dose CT scans were performed immediately prior to attenuation correction. PET data was reconstructed using VPFX-S (ordered subset expectation maximization (OSEM) using time-of-flight (TOF) and point-spread-function (PSF) corrections) with 6 iterations and 17 subsets with 3-mm smoothing, standard Z filter, and 25.6-cm field of view with a 256 × 256 matrix. List-mode data was binned into 4 × 5-min time frames, and the resulting PET images were motion corrected, summed, and co-registered to their corresponding T1-weighted MR images. Flourdeoxyglucose (FDG)-PET examination was not part of the study protocol, but a number of patients underwent clinical FDG-PET examination. If so, results from the FDG-PET were used for diagnostic purposes in the present study.

### MRI acquisition and processing

The participants were all scanned on a Siemens MAGNETOM Prisma 3.0 T MRI scanner, acquiring sagittal isometric 1-mm^3^ T1-weighted magnetization-prepared rapid gradient-echo (MP-RAGE) inversion recovery images. MR images were processed using an in-house-developed pipeline including the removal of non-brain tissue (brain extraction), segmentation into grey and white matter, parcellation into regions of interest (ROI), and normalization of images into Montreal Neurological Institute (MNI152) standard space.

### Tau PET region-of-interest definition

T1-weighted MR images were parcellated using FreeSurfer v6.0 software (https://surfer.nmr.mgh.harvard.edu/) and the Desikan Killiany atlas [20]. Using the inferior cerebellar grey matter region as the reference region, the standardized uptake value ratio (SUVR) was calculated on the corresponding PET image. Partial volume correction (PVC) was performed using the geometric transfer matrix method [21]. We used two complimentary data analysis strategies, one region-of-interest (ROI) based and one voxel based. Composite ROIs were created based on the ROIs from the Desikan Killany Atlas. In line with earlier work [15, 22, 23], bilateral regions-of-interest (ROIs) capturing areas of the brain most prominently affected by tau pathology in early and intermediate stages of AD were selected. These included (early) the entorhinal cortex [24, 25] and (intermediate) a temporal Meta-ROI comprising a weighted average of entorhinal, amygdala, parahippocampal, fusiform, and inferior and middle temporal ROIs [26]. In addition, to better capture frontal and anterior temporal regions typically affected in FTD, we created a frontal meta-ROI with weighted average of the anterior cingulate, the frontal pole and superior/orbital/caudal middle frontal, and inferior frontal cortex, and, lastly, an anterior temporal Meta-ROI comprising the entorhinal cortex and the temporal pole. In addition to the inferior cerebellar region, ROI analyses were also run using white matter as the reference region. This was defined using a subject-specific parametric estimation of reference signal intensity (PERSI) [27]. The purpose of this procedure was to ensure that unexpected binding to dipeptide repeat proteins (DRP) in the cerebellar cortex [28] did not influence the results.

### Voxel-based morphometry

In order to examine the overlap between tau PET and grey matter atrophy, voxel-based morphometry (VBM) was performed [29]. In brief, the diffeomorphic nonlinear image registration tool (DARTEL) [30] was first used to create a study-specific template using grey and white matter parcellation maps from the SPM-based preprocessing step. Once the template was created, segmented grey matter images were warped into MNI space using the individual flow fields resulting from the DARTEL registration, and voxel values were modulated for volumetric changes introduced by the normalization.

### W-score maps

For voxel-wise PET analyses, SUVR images were spatially transformed into a common MNI152 space using the transformation derived from MRI normalization and smoothed at 6 mm with a full width at half-maximum Gaussian kernel. In order to obtain images showing tau PET SUVR and grey matter density at the individual patient level, we computed W-score maps (Z-score maps adjusted for age) using SUVR and grey matter VBM images, as described elsewhere [31, 32]. Mean and standard deviation images were derived from a group of Aβ-negative CU individuals (*n* = 50; age, 71 ± 9.20 years) from the BioFINDER-2 study. Calculations were performed using SPM12 (Wellcome Department of Cognitive Neurology, London, UK; https://www.fil.ion.ucl.ac.uk/spm) in MATLAB (v. 9.2, 2017a).

### In vitro autoradiography and neuropathology

Autoradiography was performed on six FTD cases from The Netherlands Brain Bank and one case with AD using [^3^H]RO948 as an in vitro radiotracer. Description of cases, autoradiography methods, and procedure of the neuropathological examination are found in the Supplementary Material.

### Statistical analyses

Differences in baseline characteristics were assessed using Fisher’s exact test for categorical data and the Kruskal–Wallis test with post hoc Dunn’s pairwise comparison for continuous variables. Group-wise differences in [^18^F]RO948SUVR across ROIs were compared using the Mann–Whitney U test. Effect size for SUVR comparisons between ROIs was calculated as Cohen’s d, i.e., (mean_1_ − mean_2_)/SD_pooled_. Bonferroni correction was applied to account for multiple comparisons. All analyses were performed in R, version 4.0.2 (R Foundation). Significance was set at a two-sided level of *P* < 0.05.

## Results

### Participants

Demographic information, results of MMSE, and clinical dementia rating (CDR) can be found in Table 1. In total, the FTD cohort in the present study is composed of 35 individuals: 21 non-genetic probable bvFTD patients, 1 patient with non-genetic bvFTD and ALS, 11 symptomatic *C9orf72* mutation carriers, one case of bvFTD due to a *GRN* mutation (c.328C > T, R110X), and one case with bvFTD due to a *MAPT* mutation (c.1216C > T, R406W) (Table 1). Nine of the *C9orf72* patients had bvFTD as the clinical phenotype, one had MCI, and one PPA according to basic PPA criteria but that did not meet criteria for any PPA subtype [17]. Two patients fulfilled revised El Escorial criteria for clinical probable ALS [33] in addition to their bvFTD diagnosis. One of these was a *C9orf72* mutation carrier, and the remaining was negative for *C9orf72* and superoxide dismutase (*SOD*) mutations. By comparison to the other groups, the sporadic FTD group was significantly older (genetic FTD, Aβ-negative CU, AD dementia, *P* < 0.01). The FTD groups and patients with AD dementia showed significantly lower MMSE scores compared to Aβ-negative CU (*P* < 0.01). The proportion of the individuals carrying at least one APOE ε4 allele was significantly greater in Aβ-negative CU and AD dementia compared to sporadic FTD (Aβ-negative CU, *P* < 0.01; AD dementia, *P* < 0.001) and genetic FTD (AD dementia, *P* < 0.01). Two of the patients have undergone neuropathological examination (see below). All patients included did have frontal and/or temporal pathologic neuroimaging at the baseline on neuroimaging (either atrophy on MRI or FDG-PET hypometabolism) except two cases with bvFTD due to *C9orf72* mutation (normal MRI and FDG-PET) and the case with MCI due to a*C9orf72* mutation (normal MRI, no FDG-PET performed).

**Table 1:** Participant demographic and clinical characteristics

	Sporadic FTD	Genetic FTD	Aβ-negative CU	AD dementia
*N*	22	13	13	13
Age, year	72.67 (5.30)^a,b,c^	66.02 (6.14)	66.92 (5.13)	67.49 (5.16)
Sex, F/M	12/10	8/5	8/5	7/6
MMSE	25.50 (2.47)^d^	23.67 (3.77)^d^	28.68 (1.00)	21.74 (5.18)^d^
*APOE* ε4 carrier, *n* (%)	1 (4.5%)	2 (15%)	7 (58%)^e^	9 (75%)^f,g^
Aβ-positive, *n* (%)	5 (23%)	2 (22%)^*^	0 (0%)	13 (100%)
Mutation (*n*; %)	-	11 *C9orf72* (84.6%) 1 *GRN* (7.7%), 1 *MAPT* (7.7%)	-	-
Clinical subtype, *n* (%)	21 BvFTD (83%) 1 BvFTD + ALS (17%)	12 bvFTD (92%) 1 PPA (8%)	-	-
CDR	0.73 (0.26)	0.75 (0.34)	-	-
FTLD CDR SB	6.73 (2.3)	6.04 (3.08)	-	-

*AD*, Alzheimer’s disease; *Aβ-CU*, Aβ-negative cognitively unimpaired individuals; *APOE*, apolipoprotein E; *FTD*, frontotemporal dementia; *CDR*, clinical dementia rating; *FTLD CDR SB*, FTLD version of CDR, sum of boxes; *MMSE*, mini mental status examination

Aβ-positivity is according to CSF Aβ status using the Aβ42/Aβ40 ratio. ^*^ CSF data missing for 4 subjects. ^a^Significantly higher than Aβ-negative CU, *P* < 0.01; ^b^significantly higher than AD dementia, *P* < 0.01; ^c^significantly higher than genetic FTD, *P* < 0.01; ^d^Significantly lower than Aβ-negative CU, *P* < 0.01; ^e^significantly higher than sporadic FTD, *P* < 0.01; ^f^significantly higher than sporadic FTD, *P* < 0.001; ^g^significantly higher than genetic FTD, *P* < 0.01

### ROI-based analyses

**Fig. 1:**
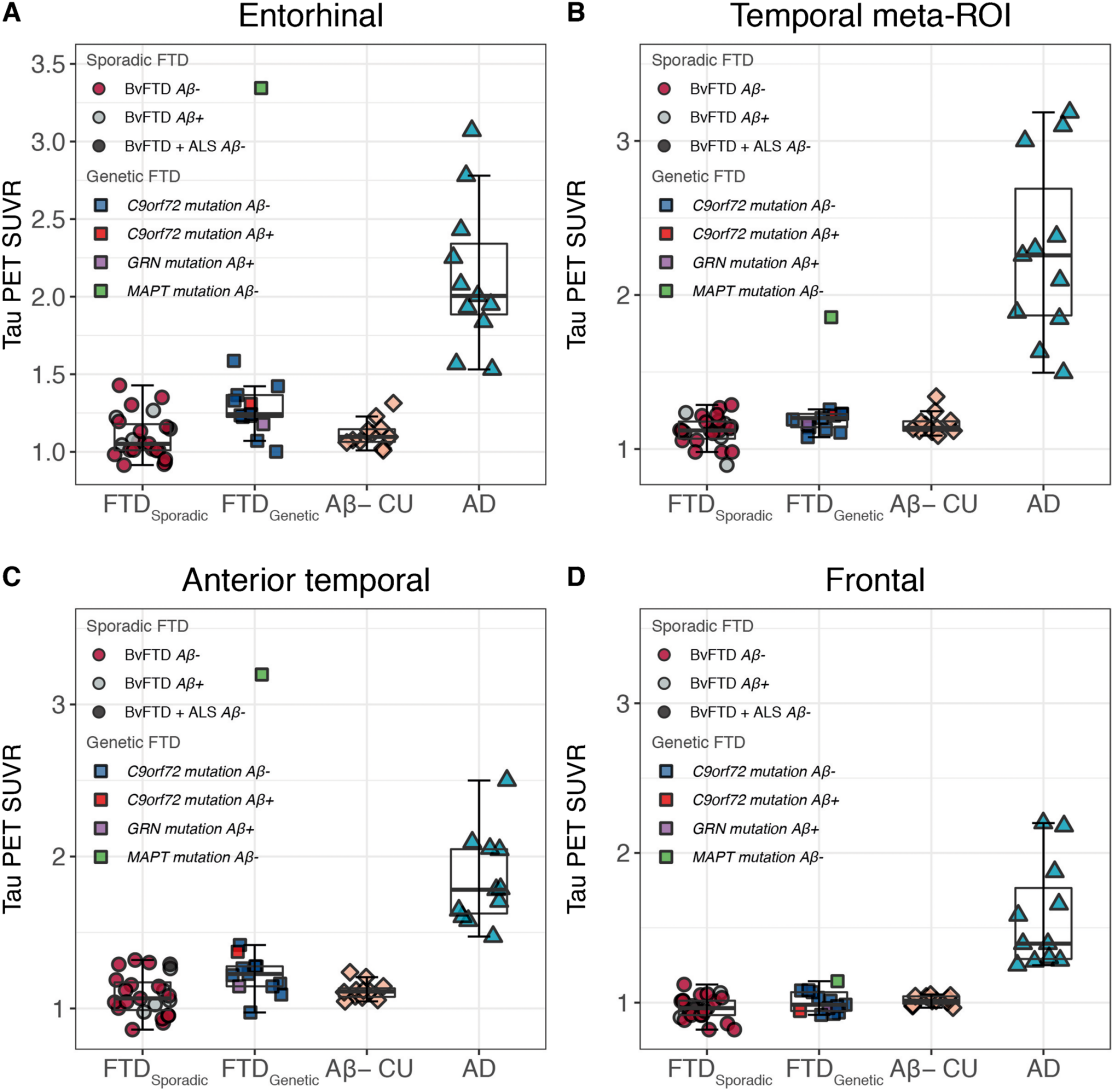
[^18^F]RO948 standardized uptake value ratios (SUVRs) across diagnostic groups in regions of interests AD: Alzheimer’s disease (*n* = 13); Aβ-CU: Aβ-negative cognitively unimpaired individuals (*n* = 13); bvFTD: behavioral variant of frontotemporal dementia (*n* = 21); bvFTD ALS: bvFTD and amyotrophic lateral sclerosis (*n* = 1), *C9orf72*: chromosome 9 open reading frame 72 (*n* = 11); *GRN*: progranulin (*n* = 1); *MAPT*: microtubule-associated protein tau (*n* = 1). No significant differences were seen in either the FTD group compared to Aβ-negative CU individuals. The patients with AD dementia had significantly higher SUVR values across all ROIs compared with Aβ-negative CU (*P* < 0.001), sporadic FTD (*P* < 0.001), and genetic FTD (*P* < 0.001). Box and whisker plots are shown overlaid over data points (box, median, and quartiles 1 and 3; whiskers, minimum and maximum). Amyloid status is based on the CSF Aβ42/40 ratio

Results of the ROI-based analyses are presented in Fig. 1 with SUVR data provided in Table 2. The temporal meta-ROI shows a complete separation of the AD cases from all Aβ negative CU (as expected) but also a complete separation of all FTD (sporadic and genetic) from all AD cases (the individual with bvFTD due to R406W*MAPT* mutation, which showed AD-like retention levels, is the exception). The mean values and clustering of the FTD cases and the Aβ negative CU cases were almost identical. The frontal meta-ROI had less tracer retention in AD cases than in the temporal meta-ROI (as expected) but still distinguished AD from Aβ negative CU and FTD with similar mean values and clustering. These two meta-ROIs cover large proportion of the cortex, including regions typically affected by protein deposition in FTD. In the entorhinal and anterior temporal Meta-ROI, the sporadic FTD cases displayed some more heterogeneity than the Aβ-negative CU cases but almost identical mean SUVR. No significant differences were seen in either FTD group compared to Aβ-negative CU individuals, including when using *p*-values uncorrected for multiple testing. The patients with AD dementia had significantly higher SUVR values across all ROIs compared with Aβ-negative CU (*P* < 0.001), sporadic FTD (*P* < 0.001) and genetic FTD (*P* < 0.01, entorhinal cortex, temporal meta-ROI, anterior temporal meta-ROI, *P* < 0.01; frontal meta-ROI, *P* < 0.001). *P*-values along with effect sizes are included in Table 2. Using the white matter as the reference region (Supplementary Fig. 1) instead of inferior cerebellar cortex for the ROI analysis did not change results in any significant manner.

**Table 2:** Regional [^18^F]RO948 tau PET SUVR

	Mean SUVR (SD)			
Groups	Entorhinal cortex	Temporal	Anterior temporal	Frontal
Sporadic FTD	1.10 (0.14)	1.12 (0.10)	1.09 (0.14)	0.96 (0.08)
Genetic FTD	1.43 (0.59)	1.23 (0.21)	1.37 (0.56)	1.01 (0.07)
Aβ-negative CU	1.11 (0.08)	1.17 (0.07)	1.13 (0.06)	1.02 (0.05)
AD dementia	2.14 (0.44)	2.36 (0.71)	1.83 (0.28)	1.55 (0.38)
Group comparisons	*P*-value (Cohen’s *d*)			
Sporadic FTD vs Aβ-negative CU	0.62 (0.17)	0.15 (0.58)	0.42 (0.26)	0.08 (0.88)
Genetic FTD vs Aβ-negative CU	0.09 (0.73)	0.55 (0.25)	0.14 (0.62)	0.66 (0.18)
AD dementia vs Sporadic FTD	< 0.001 (2.95)	< 0.001 (2.06)	< 0.001 (3.21)	< 0.001 (2.39)
AD dementia vs Genetic FTD	< 0.01 (1.31)	< 0.01 (1.89)	< 0.01 (1.05)	< 0.001 (2.24)
AD dementia vs Aβ-negative CU	< 0.001 (2.98)	< 0.001 (1.97)	< 0.001 (3.34)	< 0.001 (2.23)

Standardized uptake value ratio (SUVR) values are displayed as mean (standard deviation)

*AD*, Alzheimer’s disease; *Aβ-CU*, Aβ-negative cognitively unimpaired individuals; *APOE*, Apolipoprotein E; *FTD*, frontotemporal dementia; *ROI*, region-of-interest. The temporal meta-ROI included entorhinal, amygdala, parahippocampal, fusiform, and inferior and middle temporal gyri; the anterior temporal ROI included the entorhinal cortex and the temporal pole; the frontal meta-ROI included the anterior cingulate, the frontal pole, the superior/orbital/caudal middle frontal, and the inferior frontal. Cohen’s d was calculated as the difference between mean SUVR divided by the pooled standard deviation

### Voxel-based analyses

**Fig. 2:**
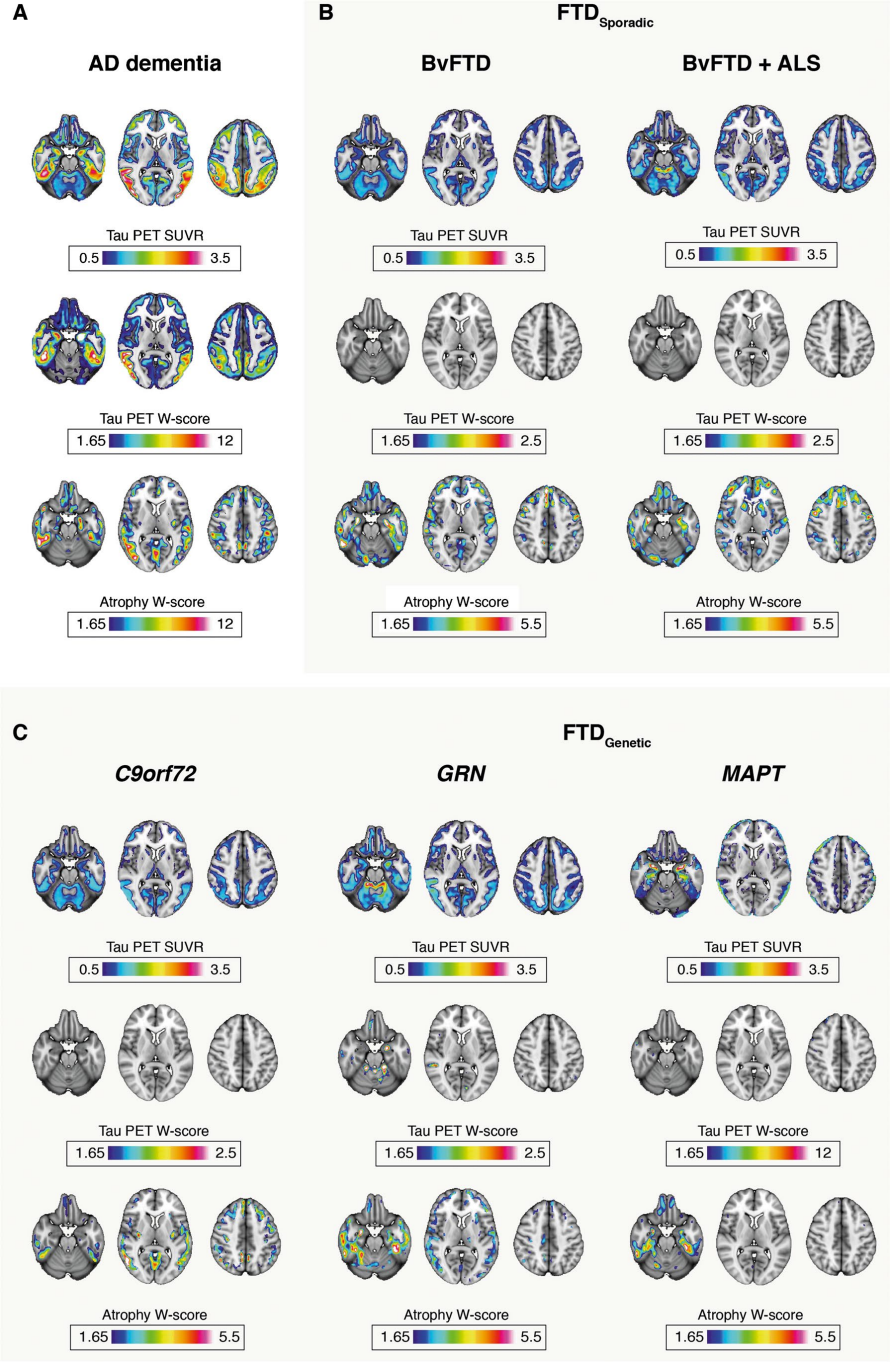
Results of the PET voxel-based analyses and voxel-based morphometry. PET results are displayed using standardized uptake value ratios (Tau PET SUVRs) and W-scores (Tau PET W-scores), and the voxel-based morphometry using W-scores (Atrophy W-scores).W-scores are displayed  ≥ 1.65, which corresponds to *p* < 0.05. Supplementary Fig. 2 displays W-scores  ≥ 0.5. AD: Alzheimer’s disease (*n* = 13); bvFTD: behavioral variant of frontotemporal dementia (*n* = 21); bvFTD ALS: bvFTD and amyotrophic lateral sclerosis (*n* = 1), *C9orf72*: chromosome 9 open reading frame 72 (*n* = 11); *GRN*: progranulin (*n* = 1); *MAPT*: microtubule-associated protein tau (*n* = 1). Please note that tau PET W-score scale bars differ for AD and *MAPT*. The MAPT case scale bar is chosen because of the AD-like retention level seen

Results of the voxel-based analyses are displayed in Fig. 2. The rationale for the voxel-based PET analyses was to capture other regions of tracer retention than the selected ROIs, to show a global picture of tracer retention, and, in the case of W-scores, to put tracer levels in relationship to controls, covarying for age. In Fig. 2, W-scores are displayed  ≥ 1.65, which corresponds to *p* < 0.05, while Supplementary Fig. 2 displays W-scores  ≥ 0.5. Figure 2A shows a classic pattern of temporo-parietal tracer binding and atrophy typical of AD. Figure 2B SUVR shows that sporadic FTD cases had a level of tracer retention throughout the cerebrum in line with that in the reference region, indicating no specific binding. The W-score analysis in FTD groups shows as whole tracer levels comparable to those in Aβ-negative CU, and no binding pattern resembling that of the cortical atrophy (as shown by the atrophy W-score analysis) emerged. The individual with bvFTD and ALS (Fig. 2B) displayed reference region levels throughout the brain with some increased SUVR levels in the superior cerebellum that, however, did not reach W-score above 1.65 and that did not correspond to cortical atrophy.

Among the genetic cases (Fig. 2C), the *C9orf72* mutation carriers appeared very similar to sporadic bvFTD with SUVR maps, showing reference region level retention and PET W-score maps at control levels. Cortical atrophy patterns were, as expected for *C9orf72*, fronto-temporo-parietal more than fronto-temporal. The entorhinal cortex and temporal pole, as indicated by the ROI analysis (Fig. 1), did not show increased retention. The individual with bvFTD due to *GRN* mutation showed largely similar results on the SUVR map as sporadic and *C9orf72* bvFTD but had loci of elevated binding in the superior cerebellum and the temporal lobe that overlapped but did not correspond to observed atrophy. The individual with *MAPT* mutation bvFTD showed a temporal dominant pattern of cortical atrophy, particularly accentuated in the medial temporal lobes, with corresponding elevated binding on SUVR and W-score maps.

### In vitro autoradiography and neuropathology

**Fig. 3:**
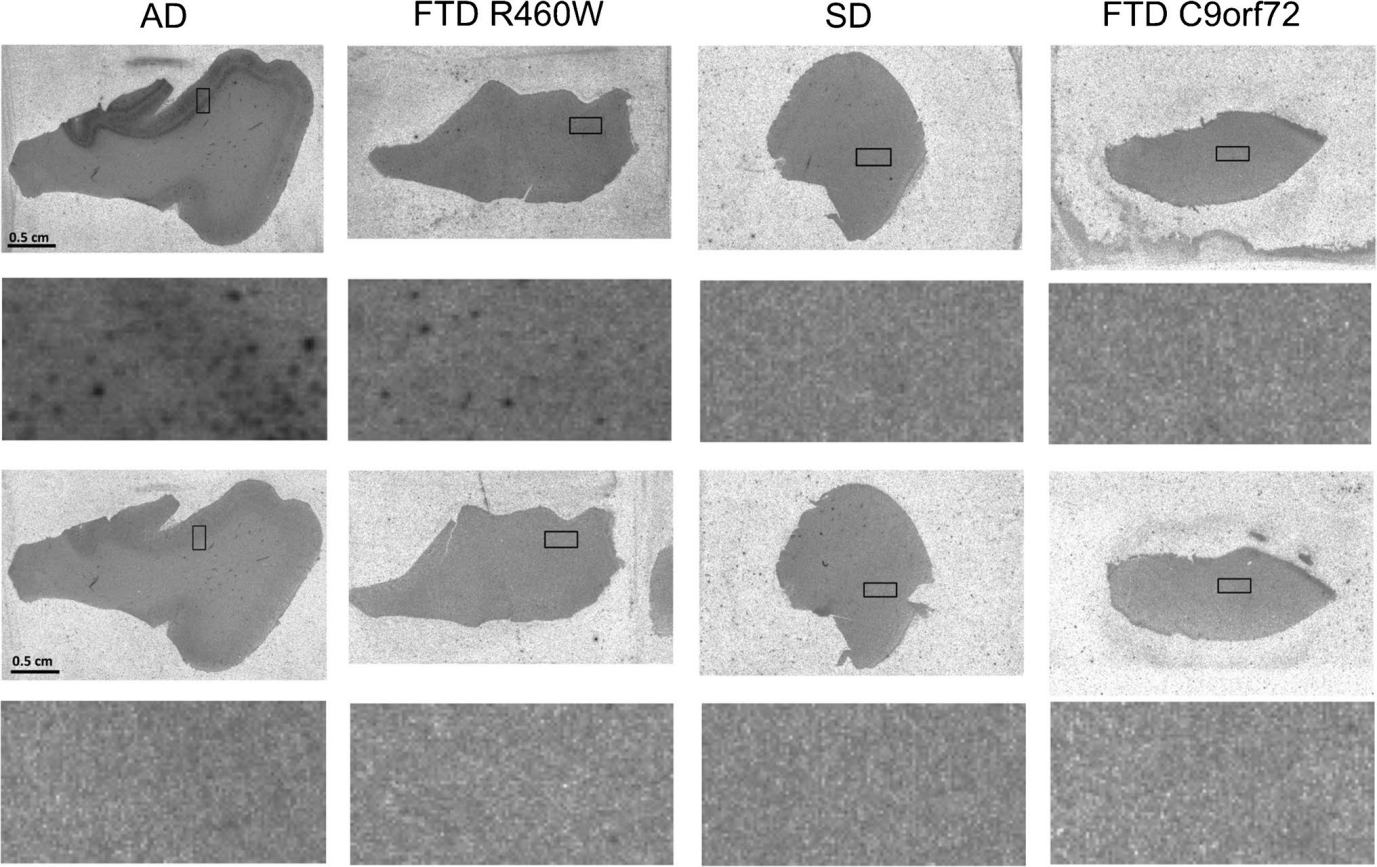
Results of the autoradiography. Total [^3^H]RO948 binding and nonspecific binding to representative brain tissue slices are shown. First row: total [^3^H]RO948 binding; second row: detail; third row: nonspecific binding; fourth row: detail. AD: superior temporal gyrus of individual with Alzheimer´s disease, Braak stage V. FTDR406W: inferior temporal gyrus of case with frontotemporal dementia due to R406W tau mutation. SD: frontal gyrus of case with semantic dementia, TDP-43 type C; FTD *C9orf72*: temporal gyrus of case with FTD due to *C9orf72* mutation, TDP-43 type B

Specific [^3^H]RO948 binding was detected in cortical tissue sections from the two cases with FTD due to R406W *MAPT* mutation and the case with AD (Fig. 3), while specific binding was neither seen in tissue samples from semantic dementia (SD) cases (TDP-43 type C and TDP-43 unspecified subtype) nor in the cases with bvFTD due to *C9orf72* (TDP-43 type B) (Supplementary Table 1). Radioligand binding colocalized with AT-8 antibody staining of tau aggregates in FTD due to R406W *MAPT* mutation (Supplementary Fig. 3). Neuropathological examination postmortem was performed in two cases with *C9orf72*-associated bvFTD and is described in detail in the Supplementary Material. Both cases showed neurodegeneration with TDP-43 positive pathology (not identical to but most closely resembling TDP-43 type A), cerebral atrophy while no significant in vivo tracer binding (Supplementary Figs. 4 and 5).

## Discussion

The aim of the current study was to examine the binding of the tau PET tracer [^18^F]RO948 in FTD in a large sample of the heterogenous molecular pathologies underlying the syndrome of FTD, enriched with cases with a strong clinicopathological relationship. Overall, there appears to be no specific binding of [^18^F]RO948 in FTD, except in *MAPT* mutation carriers (depending on the particular mutation). The following discussion will be structured around the presumed molecular pathologies.

### C9orf72 mutation carriers (presumably TDP-43 A or B)

*C9orf72* molecular pathology is almost invariably TDP-43 [5, 6]. TDP-43 type B is seen when the phenotype is FTD-ALS, whereas bvFTD *C9orf72* can be TDP-43 type A or a mix of TDP-43 A and B [34, 35]. Our finding from FTD cases due to *C9orf72* mutations, the autoradiography of TDP-43 type B cases, and the absence of tracer retention in the two TDP-43-positive proven cases (similar to TDP-43 type A) speaks against [^18^F]RO948 binding to TDP-43 A and B. Though some signal was seen in the medial temporal lobes in the ROI analysis, our neuropathological examinations and the literature show a clear propensity of TDP-43 inclusions in the frontal cortices [36] and not for the medial temporal lobes [36, 37].

Low degrees of RO948 binding to TDP-43, thus, do not seem a likely answer for the medial temporal binding in the ROI analysis. p62-positive, TDP-43-negative DRPs are a feature of *C9orf72* FTD, characteristically present in the medial temporal lobe and in the cerebellum [28]. However, DRP pathology is still, by magnitudes, most abundant in the frontal cortex, which makes RO948 binding to DPR less likely as an explanation. Though the ROI findings could be explained by comorbid AD, the five individuals with highest retention were all but one amyloid negative (Fig. 1A). Also, the cases that underwent neuropathology had no medial temporal lobe tracer binding (Supplementary Fig. 5) despite being Braak stages I and II, indicating that [^18^F]RO948 is likely unable to detect early pathological stages of neurofibrillary tangles, similar to what has been shown for [^18^F]flortaucipir [38].

The preclinical work (including autoradiography) in [^18^F]RO948 [39, 40] has not pointed to any other binding target in the CNS that could explain our findings. The tau PET tracer [^18^F]flortaucipir has been used to study *C9orf72* FTD, showing practically no binding [13] or binding in between AD and controls [14]. [^18^F]Flortaucipir autoradiography has shown no binding to TDP A, B, or C [13, 41], but in one *GRN* case with autopsy [32] binding corresponded well to areas of TDP-43 type-A deposition. This remains to be explained, but, possibly, there could be low-level binding in vivo that does not remain in vitro [32]. The results in our present study point to [^18^F]RO948, having no specific binding to *C9orf72* FTD/TDP-43 type A nor B, and suggest that the binding might be lower than that in [^18^F]flortaucipir. To demonstrate this, head-to-head comparisons would be necessary.

### GRN mutation carrier (presumably TDP-43 type A)

In the bvFTD case due to *GRN* mutation, voxel-wise analysis showed loci with W-scores  ≥ 1.654, but the spatial pattern did not match that of atrophy in this individual, nor atrophy in *GRN* FTD cases generally [42]. Neither did the loci match the known pathology distribution in FTD *GRN*, which all is mostly frontally dominant and does not particularly affect the cerebellum [43]. In a case of bvFTD due to *GRN* mutation reported by Tsai and coworkers, [14] [^18^F]flortaucipir binding was elevated compared with that in controls in expected areas and mirrored cortical atrophy on MRI. In concordance, another case [32] with TDP-43 type-A inclusions due to *GRN*, [^18^F]flortaucipir binding corresponded with an inclusion pattern at neuropathology. We argue that our findings suggest no specific [^18^F]RO948 binding to *GRN*TDP-43 type A pathology and, possibly, that [^18^F]RO948 is more specific than [^18^F]flortaucipir in this regard. Again, direct comparisons of the tracers in the same individuals would be needed to demonstrate this.

### BvFTD ALS, sporadic (presumably TDP-43 type B)

This case showed scattered loci of [^18^F]RO948 binding but none  ≥ 1.65 on W-scores and with no pattern corresponding to the atrophy in that case, nor to bvFTD ALS atrophy in general nor to the distribution of TDP-43 type B inclusions in FTD-ALS cases, which is dominant in frontal, temporal, and motor cortex [28]. Together with the autoradiographic results on TDP-43 type B, these findings suggest no specific RO948 binding to TDP-43 type B. The literature on other tau tracers in cases with presumed TDP-43 type B is very limited. Soleimani-Meigooni et al. (2020) [32] showed increased [^18^F]flortaucipir SUVR in frontal white matter, which did not match neither the extensive TDP-43 staining cortically frontally nor the minor frontal tau staining seen in one TDP-43 type-B case that underwent autopsy. Tsai et al. [14] report a *C9orf72* case with ALS and executive dysfunction with some increased binding in the temporal lobes, but not as expected in the motor or frontal cortex. Although the available data is limited, it appears that [^18^F]RO948 performs similar as [^18^F]flortaucipir regarding TDP-43 type B and shows no specific binding.

### SvPPA (presumably TDP-43 type C)

SvPPA, representing TDP-43 type C pathology, has shown [^18^F]flortaucipir binding in vivo corresponding to its characteristic anterior temporal cortical atrophy [10, 13, 44]. Though svPPA cases were not examined in vivo in the current work, the [^3^H]RO948 autoradiography in svPPA TDP-43 type C and svPPA TDP-43 (unspecified type) were clearly negative. Negative autoradiography stain is, however, the case also in [^18^F]flortaucipir, despite the aforementioned in vivo findings[10, 13, 44]. This indicates that it is not possible to draw conclusions on [^18^F]RO948 in vivo based on the autoradiography findings. Our previous in vivo [^18^F]RO948 study on 7 svPPA individuals [15] has shown retention in svPPA similar to that in controls. In three individuals who underwent scanning with both [^18^F]RO948 and [^18^F]flortaucipir, from the same study [15], voxel-wise subtraction analysis showed higher cortical binding in [^18^F]flortaucipir compared with [^18^F]RO948. A recent study using [^18^F]THK5351 [45] has also shown retention in svPPA, although not at AD levels. From a specificity perspective, [^18^F]RO948, thus, might be favorable in svPPA/TDP-43 type C compared to [^18^F]flortaucipir and [^18^F]THK5351. Taken together with the findings of the current study, this suggests that there is no specific in binding of [^18^F]RO948 in any of the major TDP-43 types (A, B, and C). To our knowledge, there are no tau PET studies addressing limbic-predominant age-related TDP-43 encephalopathy (LATE). TDP-43 inclusions in LATE are similar to those seen in TDP-43 type A [46], but LATE can be clinically challenging to separate from AD [47]. The specificity of [^18^F]RO948 suggested in the current study thus appears promising in this regard.

### MAPT mutation carrier (combination of 4 and 3 repeat tau)

Most *MAPT* mutations lead to the formation of neurofibrillary tangles, but which tau isoform is the main constituent of the aggregates varies across mutations and can also show variation between kindreds with the same mutation. R406W mutations lead to accumulation of both 3 and 4 repeat tau, although with 4 repeat dominance in some reports [48]. As expected, levels of RO948 binding in the case presented here are AD like and follow the expected temporal dominant pattern of R406W cases both in the ROI and voxel-wise analyses [48]. This pattern was also seen verified by specific binding in the autoradiography, which also co-localized with AT8 tau immunostaining. Previous work using [^18^F]flortaucipir has shown cortical binding in R406W mutations with both FTD and CBD phenotypes [14, 15, 49].

### Sporadic bvFTD (4 repeat tau, TDP-43, other)

In the absence of neuropathology, the protein pathology of the included sporadic bvFTD cases is unknown. Based on what pathology is behind the bvFTD syndrome generally [4, 5, 8, 50] and the sample size, we very likely have TDP-43 (between 32 and 55% of bvFTD cases, mix of type A/B/C) and tau molecular pathology represented (35–45% of bvFTD cases). The majority of tau pathology in the series would (also an estimate from the literature) be 4 repeat tau (i.e., PSP or CBD pathology), while pure 3 repeat tau (i.e., Picks disease, 7%) is rarer. This is also the case of FUS (8–13%) pathology. [^18^F]RO948 binding in our sample of sporadic bvFTD was largely similar to that in controls at the individual patient level, with only very minor loci of increased tracer binding in the (group level) voxel-based analysis that did not mirror the known distribution of protein pathology in FTD [4, 5]. All sporadic bvFTD cases had manifest frontal and/or anterior temporal cortex pathology on clinical neuroimaging, making false negative findings unlikely. Results from the present study suggest that there is no specific binding of [^18^F]RO948 in 4 repeat tau diseases, which, based on literature estimates, should be represented in our sample. This is in line with our previous results [15], showing no cortical [^18^F]RO948 retention in 16 cases of clinically diagnosed PSP, a condition with a high clinicopathological correlation to 4 repeat tau pathology [51]. To our knowledge, there are no preclinical studies on any “pure” 4 or 3 repeat tauopathy in [^18^F]RO948. The first-generation tau tracer [^18^F]flortaucipir has a problematic off-target bindings in regions affected by PSP pathology [9, 32]. There are group level differences for [^18^F]flortaucipir between PSP and controls, but not in a magnitude that can suffice for any clinical use [9, 32]. This is in contrast, however, to the situation in the 4 repeat tau condition of CBD, where studies using [^18^F]flortaucipir have shown a binding clearly different from that in controls, corresponding to affected areas, also at neuropathology [14, 32, 52, 53]. In line with this finding, half of sporadic bvFTD cases in a recent study showed [^18^F]flortaucipir retention in affected cortical areas, although only weak and spatially different from that seen in AD [14]. Novel tau tracers aimed to target at non-Alzheimer tauopathies (such as [^18^F]PI-2620) are rapidly emerging [2, 54], while struggling with problematic overlap between the regions with expected tau accumulation and localization of possible off-target binding (for a review, see [2]).

### Strengths and limitations

This is the first time where [^18^F]RO948 is reported in FTD due to mutations in *C9orf72* and *GRN*, and in FTD ALS. Together with the autoradiography cases of svPPA/TDP-43 type C, the study provides a broad palette of TDP-43 disorders. We also included a substantial number of sporadic patients to cover the most common protein pathologies. Importantly, all cases were symptomatic, all had clinical imaging indicating frontal and/or temporal atrophy and/or hypometabolism, and the vast majority were ill at a level of dementia. Thus, our cases should have representative and distributed protein pathology, minimizing the risk of false negative findings. The autoradiographical findings strongly validate our findings for pathologies with both in vitro and in vivo data (*C9orf72* and *MAPT*). The lack of neuropathology beyond two cases is the most pertinent limitation of the current study. Several of the FTD variants included show a very high (> 90%) clinicopathological correspondence, but it is not 100%, and, in addition, there is an expected overlap between pathologies [6]. Also, not all clinical FTD syndromes are represented in the current work (most notably nfvPPA; svPPA is only presented in vitro) and not the less-common genetic mutations causing FTD. Amyloid status and results of previous tau PET examinations were explicitly not an exclusion criterion. Despite this ambition, there is a possibility that results of previous examinations could influence recruitment from the clinic and thus lead to circularity.

## Conclusion

In summary, the results of the present study showed an absence of specific binding of the tau PET tracer [^18^F]RO948 in FTD, the clear exception being FTD due to R406W*MAPT* mutation. Taken together with previous work using [^18^F]RO948 [15], the current study suggests that [^18^F]RO948 may have lower binding in TDP-43 types A, B, and C, and 4 repeat tau conditions compared to [^18^F]flortaucipir, the current benchmark of tau PET tracers; further head-to-head work is needed to verify this. Similar to other tau PET tracers, [^18^F]RO948 is likely of limited utility at an individual-patient level in FTD in determining underlying neuropathology but could be useful in separating FTD from AD.

## Supplementary Information

ESM 1 (JPG 1932 kb)

ESM 2 (PNG 4660 kb)

High Resolution Image (TIF 58058 kb)

ESM 3 (PNG 866 kb)

High Resolution Image (TIFF 4423 kb)

ESM 4 (JPG 1917 kb)

ESM 5 (PNG 1458 kb)

High Resolution Image (TIF 21309 kb)

ESM 6 (DOCX 26 kb)

## Data Availability

Anonymized data will be shared by request from a qualified academic investigator for the sole purpose of replicating procedures and results presented in the article if data transfer is in agreement with EU legislation on the general data protection regulation and decisions by the Ethical Review Board of Sweden and Region Skåne, which should be regulated in a material transfer agreement.
