# Supplementary figures and images for: [18F]RO948 tau positron emission tomography in genetic and sporadic frontotemporal dementia syndromes
Source: Eur J Nucl Med Mol Imaging. 2022 Dec 14;50(5):1371–83. doi: 10.1007/s00259-022-06065-4 (PMC10027632; doi:10.1007/s00259-022-06065-4)

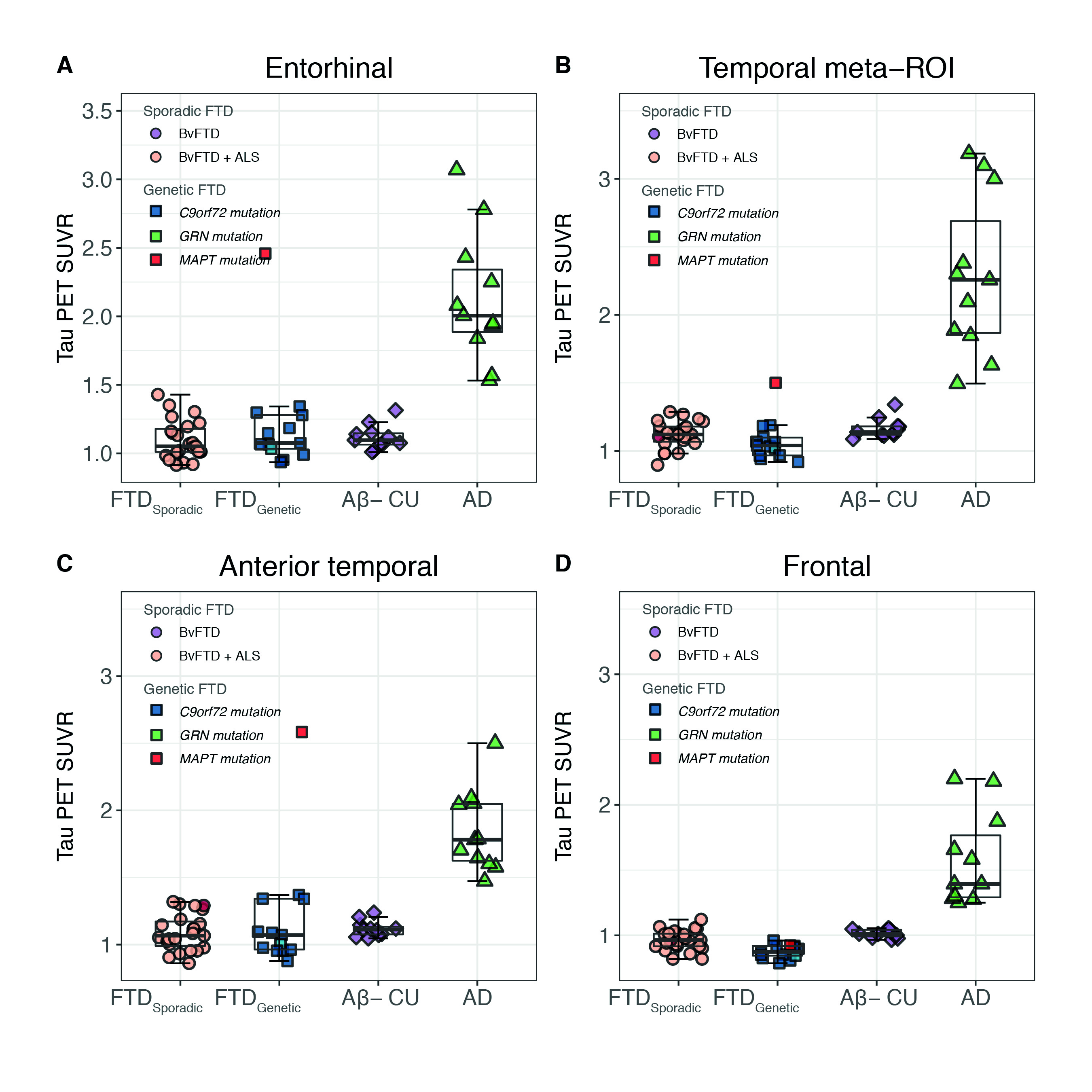

Supplement: Supplementary file 1 — (JPG 1932 kb) [file 259_2022_6065_MOESM1_ESM.jpg]

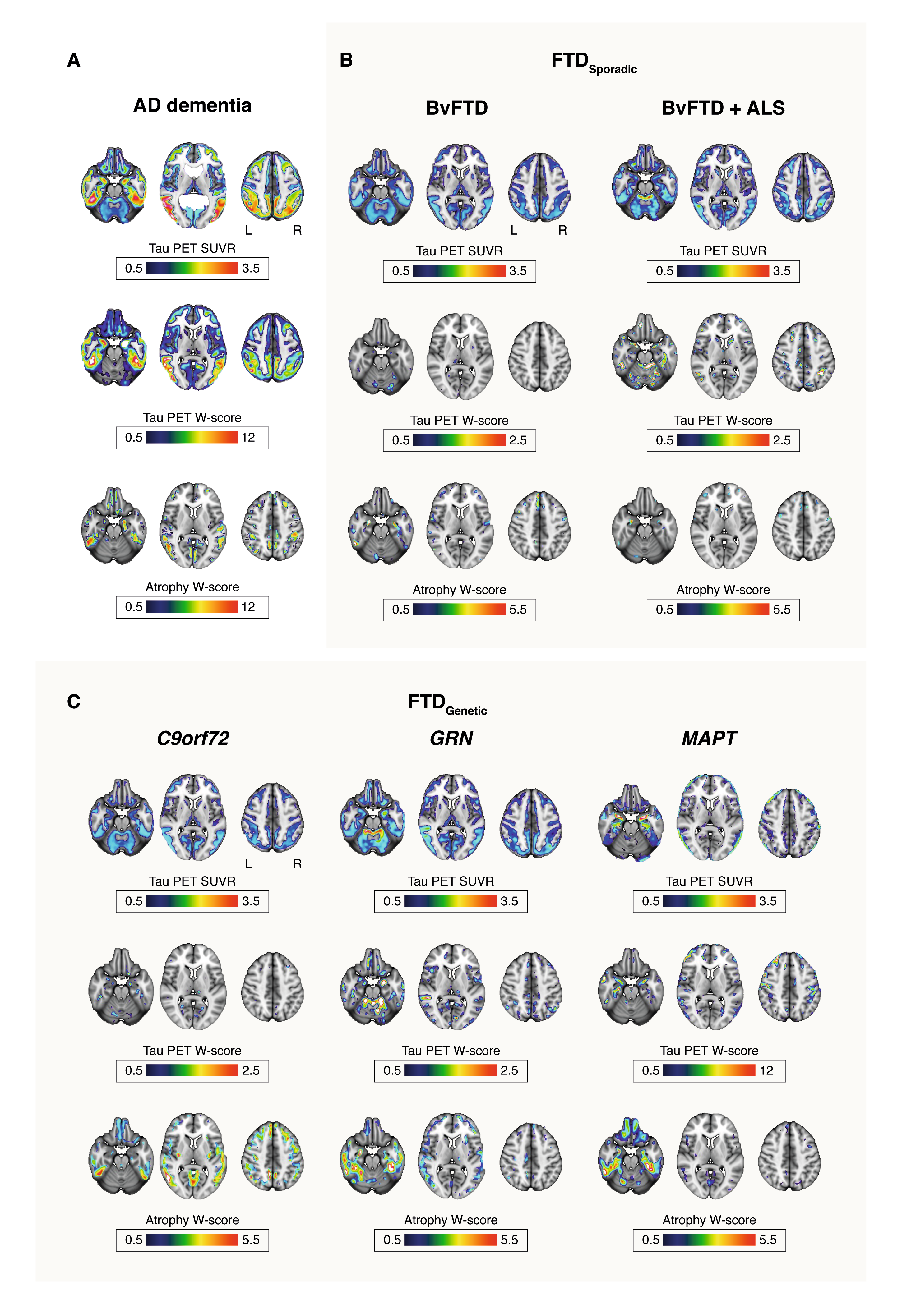

Supplement: Supplementary file 2 — (PNG 4660 kb) [file 259_2022_6065_Fig4_ESM.png]

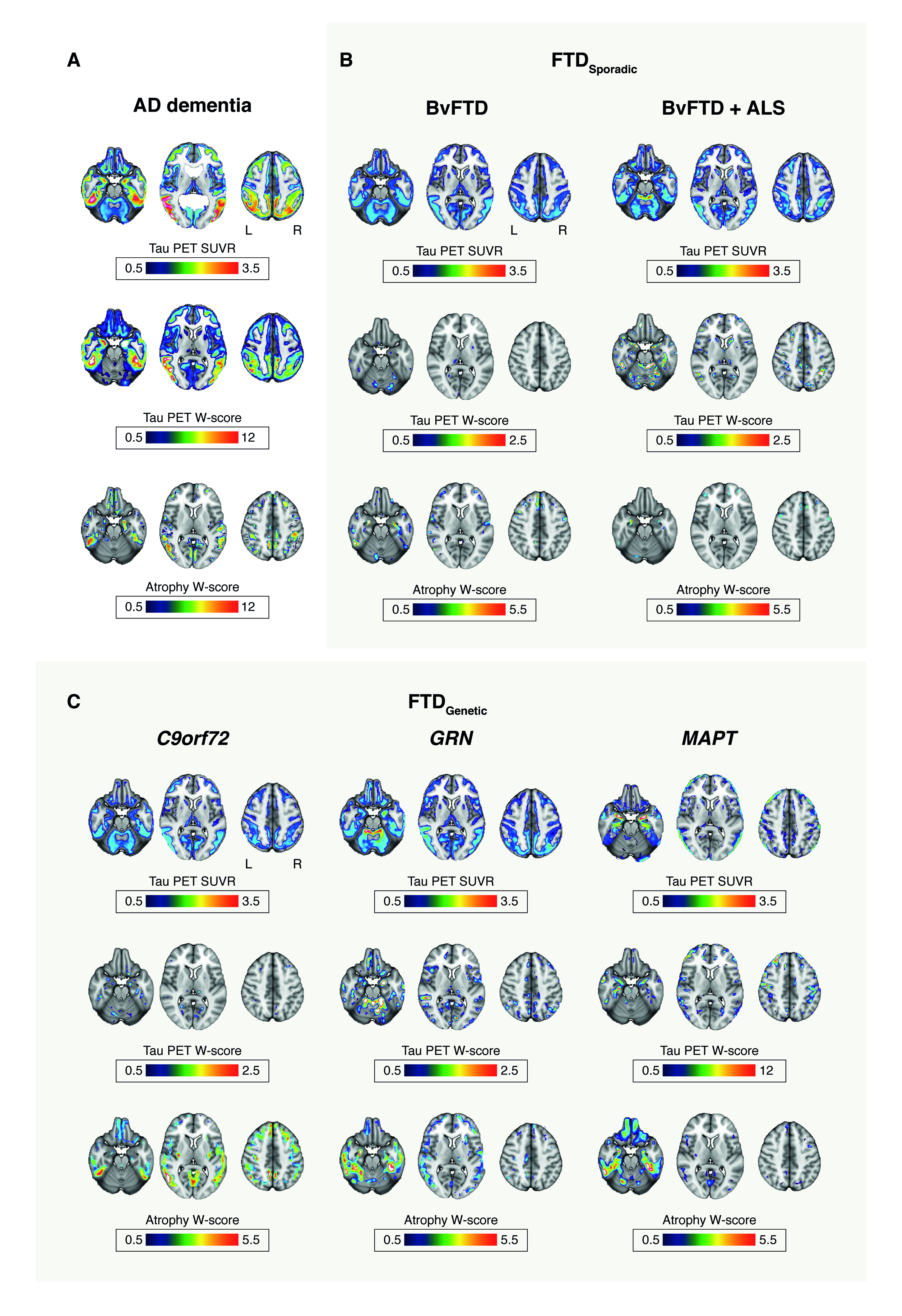

Supplement: Supplementary file 3 — High Resolution Image (TIF 58058 kb) [file 259_2022_6065_MOESM2_ESM.tif]

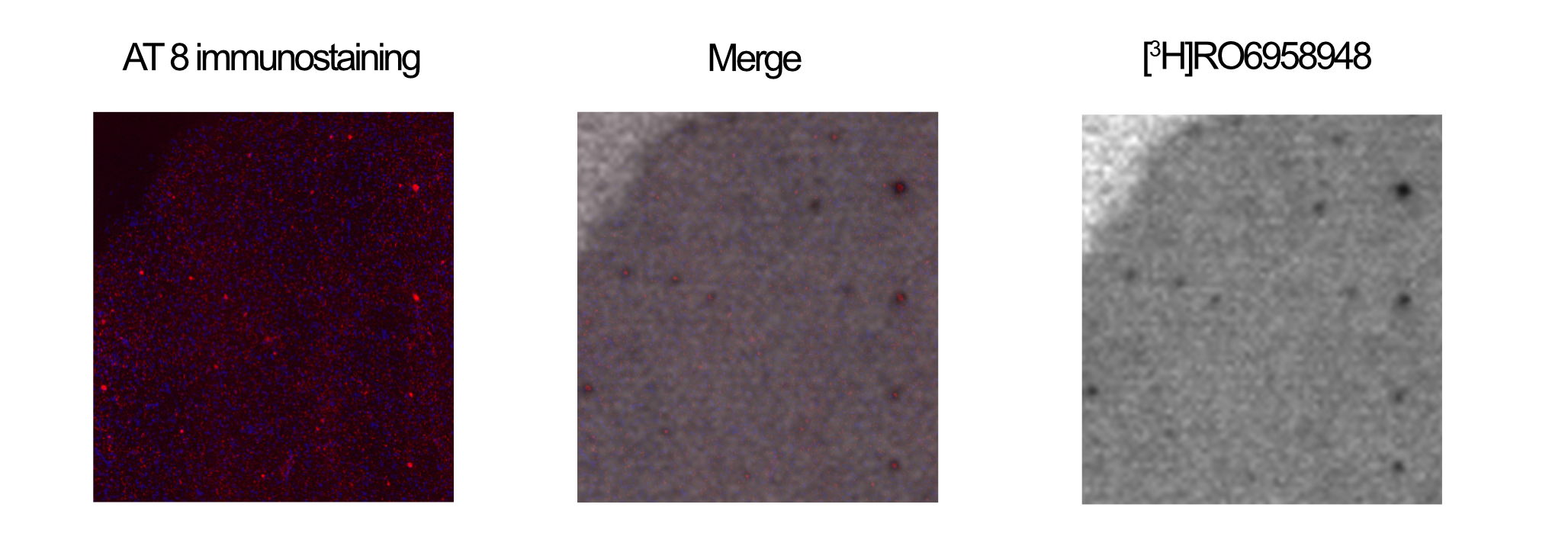

Supplement: Supplementary file 4 — (PNG 866 kb) [file 259_2022_6065_Fig5_ESM.png]

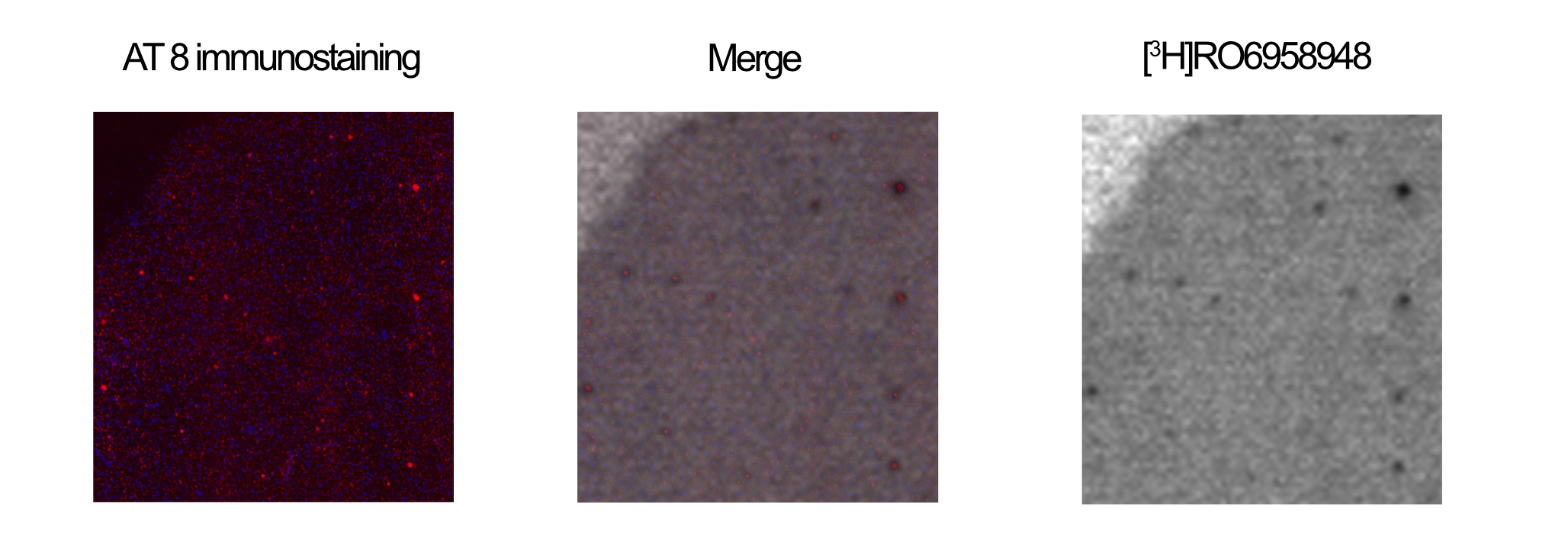

Supplement: Supplementary file 5 — High Resolution Image (TIFF 4423 kb) [file 259_2022_6065_MOESM3_ESM.tiff]

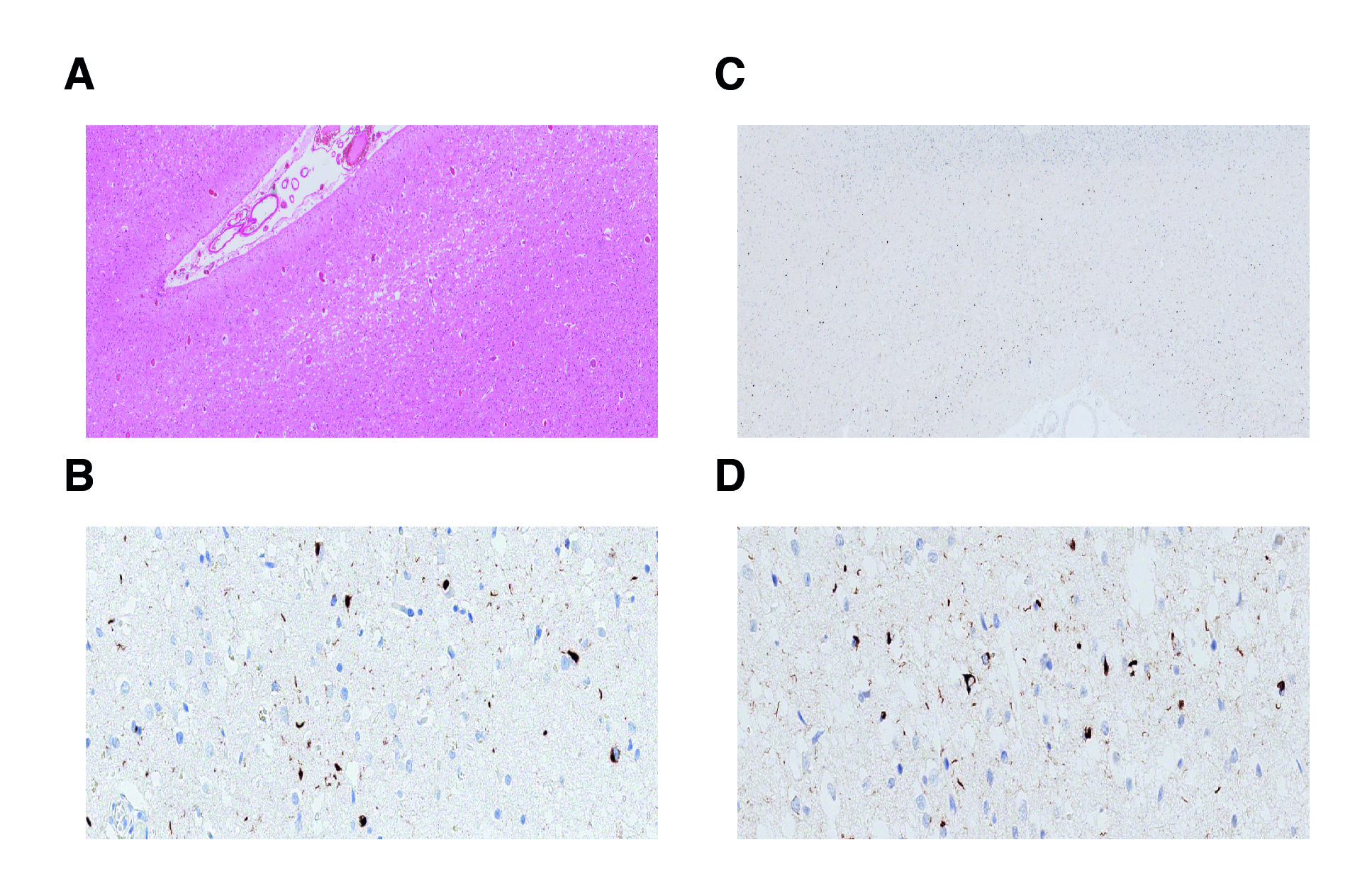

Supplement: Supplementary file 6 — (JPG 1917 kb) [file 259_2022_6065_MOESM4_ESM.jpg]

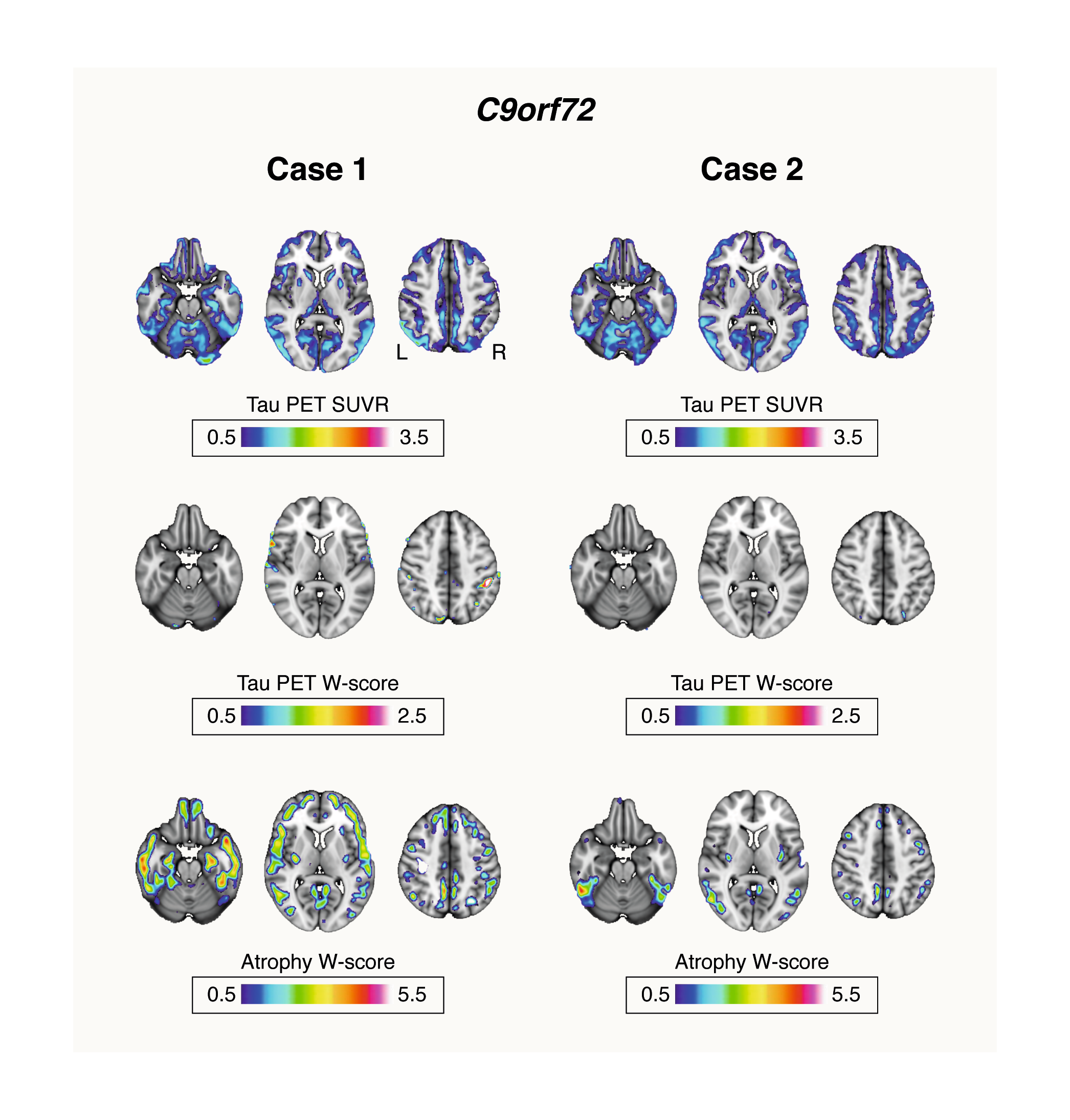

Supplement: Supplementary file 7 — (PNG 1458 kb) [file 259_2022_6065_Fig6_ESM.png]

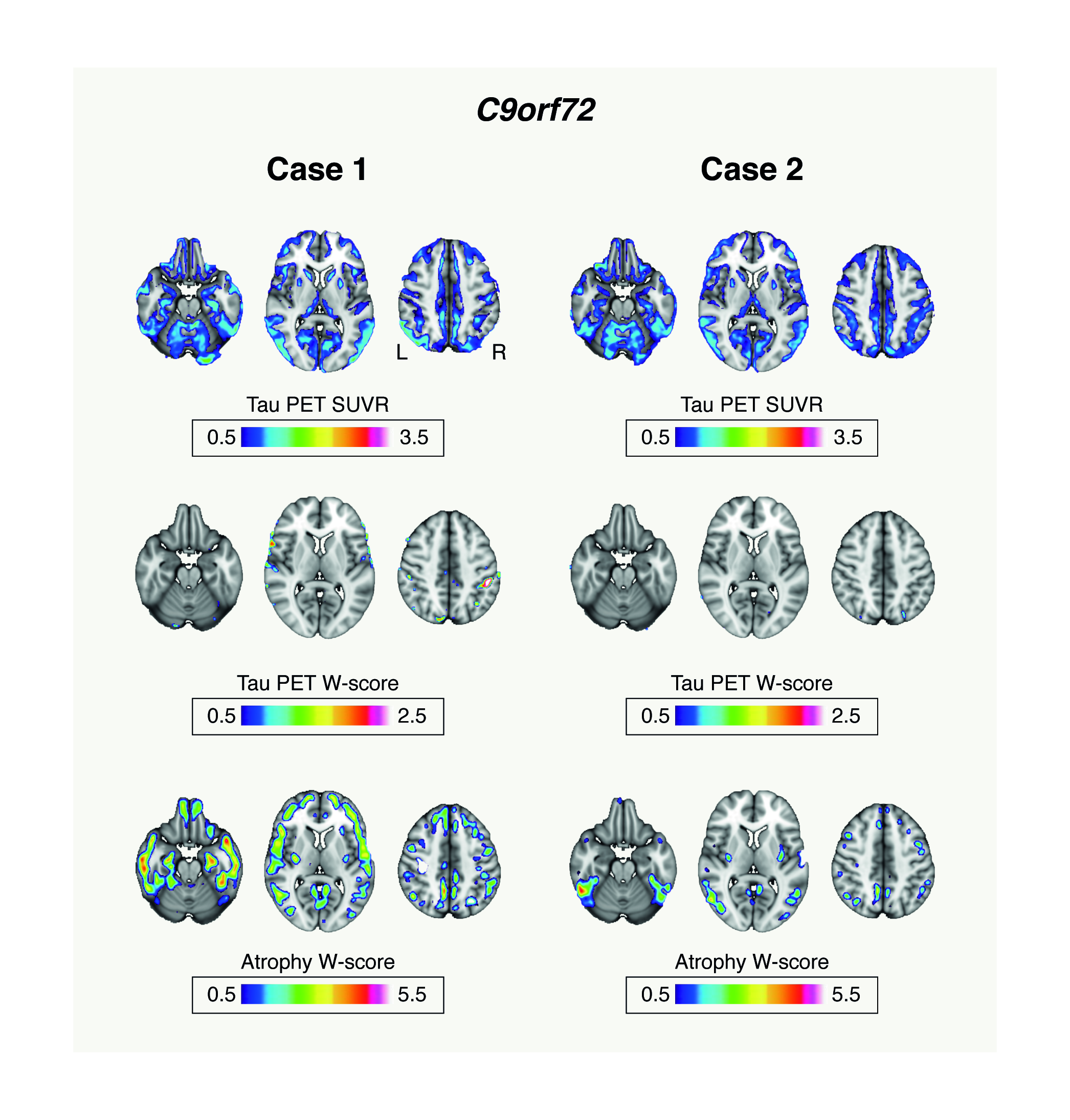

Supplement: Supplementary file 8 — High Resolution Image (TIF 21309 kb) [file 259_2022_6065_MOESM5_ESM.tif]
