# Supplementary material for: [18F]RO948 tau positron emission tomography in genetic and sporadic frontotemporal dementia syndromes
Source: Eur J Nucl Med Mol Imaging. 2022 Dec 14;50(5):1371–83. doi: 10.1007/s00259-022-06065-4 (PMC10027632; doi:10.1007/s00259-022-06065-4)
Supplement: Supplementary file 9 — (DOCX 26 kb) [file 259_2022_6065_MOESM6_ESM.docx]

**Supplementary Material**

**Title**

[^18^F]RO948 Tau Positron Emission Tomography in Genetic and Sporadic Frontotemporal Dementia Syndromes

**Running title**

RO948 PET in FTD

**Authors**

Alexander Santillo^1*^, Antoine Leuzy^1*^, Michael Honer^2^, Maria Landqvist Waldö^3^, Pontus Tideman^1^, Luke Harper^1^, Tomas Ohlsson^4^, Svenja Moes^2^, Lucia Giannini^5^, Jonas Jögi^6^, Colin Groot^1^, Rik Ossenkoppele^1,7^, Olof Strandberg^1^, John van Swieten^5^, Ruben Smith^1, 9^, Oskar Hansson^1,10^

^*^ Contributed equally

**Affiliations**

^1^Department of Clinical Sciences, Clinical Memory Research Unit, Faculty of Medicine, Lund University, Lund/Malmö, Sweden

^2^Pharma Research and Early Development, Roche Innovation Center Basel, F. Hoffmann-La Roche, Basel, Switzerland

^3^Clinical Sciences Helsingborg, Department of Clinical Sciences Lund, Lund University, Lund, Sweden

^4^Radiation Physics, Skane University Hospital, Sweden

^5^Alzheimer Center, Department of Neurology, Erasmus Medical Center, Rotterdam, The Netherlands

^6^Clinical physiology, Department of Clinical Sciences Lund, Lund University, Sweden

^7^Alzheimer Center Amsterdam, Department of Neurology, Amsterdam Neuroscience, Vrije Universiteit Amsterdam, Amsterdam UMC, Amsterdam, The Netherlands

^9^Department of Neurology, Skåne University Hospital Lund, Sweden

^10^Memory Clinic, Skåne University Hospital, Malmö, Sweden

**Corresponding author**

Alexander Santillo, MD PhD

Memory Clinic, Skåne University Hospital,

SE-20502 Malmö, Sweden

[alexander.santillo@med.lu.se](mailto:alexander.santillo@med.lu.se)

**Supplementary Methods**

***In vitro autoradiography***

Fresh frozen cortical tissue blocks (from frontal, temporal or occipital lobes) from two FTD R406W cases (six blocks in total), two semantic dementia (SD) TDP-43 positive cases (five blocks in total), two cases with FTD due to *C9orf72* mutations (TDP-43 positive cases, Type B) case (five blocks in total) were obtained from The Netherlands Brain Bank. A superior temporal gyrus block with high tau load from a late stage AD patient (89y, female, Braak V) was obtained from the Banner Sun Health Research Institute (Phoenix, USA). Ten µm thick sections of the brain tissue blocks were generated with a cryostat (Leica CM3050) at -17 °C chamber temperature and -15 °C object temperature. Sections were transferred to Histobond+ microscope slides (Marienfeld Laboratory Glasware). After drying for three hours at room temperature the sections were stored at -20 °C.

**Supplementary Table 1. Cases in the autoradiographical examination**.

| Case | Age | Gender | Clinical dx | Neuropathological dx | Tau |
| --- | --- | --- | --- | --- | --- |
| 1 | 89 | F | AD | AD Braak stage V | Positive |
| 2 | 71 | F | FTD, R406W mutation | AD Braak V-VI | Positive |
| 3 | 75 | F | FTD, R406W mutation | FTLD, tau-positive | Positive |
| 4 | 63 | M | SD | TDP-43 Type C | Negative (AT8) |
| 5 | 69 | M | SD | TDP-43, unspecified | Negative (AT8) |
| 6 | 68 | F | bvFTD, *C9orf72* mutation | TDP-43 Type B | Some nft |
| 7 | 64 | M | bvFTD, *C9orf72* mutation | TDP-43 Type B | Negative (AT8) |

[^3^H]RO948 was tritiated at Roche with a molar activity of 25.3 Ci/mmol and a radiochemical purity higher than 91%. The brain tissue sections were incubated with the radioligand (10 or 30 nM) in 50 mM Tris-HCl buffer pH 7.4 at room temperature for 30 min. After washing 3x 10 min at 4 °C in 50 mM Tris-HCl buffer pH 7.4 and three quick dips in H_2_O dist. at 4 °C, the sections were dried at 4 °C for 3 h. The sections were placed in a FujiFilm Cassette (BAS 2025), exposed to a FujiFilm Imaging Plate (BAS-IP TR 2025) for five days and afterwards scanned with a FujiFilm IP reader (BAS-5000) with a resolution of 25 µm per pixel. The autoradiograms were visualized with the software MCID analysis (version 7.0, Imaging Research Inc.). Non-specific binding of [^3^H]RO948 was assessed by co-incubation with 10 µM unlabeled T808 [1], another PET tau ligand. T808 blocks any specific binding of [3H]RO948 to tau aggregates leaving non-specific [3H]RO948 binding to tissue constituents such as lipids. The presence of tau aggregates was assessed on adjacent sections using the tau-specific antibody AT8 conjugated with Alexa555 (5 µg/mL).

***Neuropathological examination***

Two participants underwent a standardized post-mortem assessment at the Division of Pathology, Department of Clinical Sciences, Lund University. Pathological assessment was performed blinded to imaging data. The fresh brains were fixed, and tissue blocks were obtained from neuroanatomical regions of interest, including all cortical lobes, basal ganglia, thalami, brainstem and medial temporal lobes. Histological changes loss were assessed on haematoxylin-and-eosin stained sections. Immunohistochemistry was performed using antibodies against hyperphosphorylated tau (AT-8, Innogenetics, Gent, Belgium), phosphorylated TDP-43 (Proteintech, Rosemont, IL, USA) and amyloid β (MilliporeSigma, Burlington, Massachusetts, USA).

**Supplementary Results**

***Neuropathology Case 1***

Case 1 was a female patient suffering from bvFTD due to a *C9orf72* mutation who died at the age of 67 with 5 years of disease duration, 23 months after [^18^F]RO948 imaging. Mild neurodegenerative changes of gliosis and neuronal loss in superficial layers were present in frontal, temporal and parietal cortices (Suppl. Fig 3A), matching the MRI based atrophy results (Suppl Fig 4). Staining for amyloid and tau were negative, apart from tau positive neurites the entorhinal cortex and hippocampus corresponding to Braak stage II [2]. RO948 binding was also largely negative, including the medial temporal lobes (Suppl. Fig 4). TDP-43 positivity was seen to a moderate degree in frontal and parietal cortices (Suppl. Fig 3B), apparent in dystrophic neurites and neuronal cytoplasmatic inclusions in all cortical layers, a pattern that was most similar (but not corresponding entirely) to the TDP-43 type A of Mackenzie et al 2011 [3]. TDP-43 positivity was also present in the fascia dentata of the hippocampus and in the basal ganglia.

***Neuropathology Case 2***

Case 2 was a male patient suffering from bvFTD due to *C9orf72* mutation who died at 55 years of age after a disease duration of 7 years. A neuropathological examination was performed 24 months after [^18^F]RO948 examination. Neurodegenerative changes with neuronal loss and pyknosis, microvacuolisation and mild gliosis in superficial layers were present in all examined cortices, clearly more accentuated frontally where the changes were severe (Suppl. Fig. 3C). This frontal accentuation was however not seen in the W-score analysis (Suppl. Fig 4) which showed moderate atrophy frontoparietally. Tau staining was negative apart from in the entorhinal cortex (Braak stage I). There was no [^18^F]RO948 binding, specifically not in the medial temporal lobes. TDP-43 staining was present in all cortices to a generally mild degree, except for a marked protein deposition in the frontal cortex bilaterally, mirroring the morphological changes. A relatively strong TDP-43 positivity was also present in the basal ganglia and hippocampi. The TDP-43 staining showed intracellular (mostly intraneuronal) inclusions and scattered short neurites (Supplementary Figure 3D), a pattern that was most similar (but not corresponding entirely) to the TDP-43 type A of Mackenzie et al 2011 [3].

**Supplementary Fig. 1 [^18^F]RO948 Standardized Uptake Value Ratios (SUVRs) across diagnostic groups in regions of interests (ROIs)** In this analysis, white matter was used as a reference region instead of the inferior cerebellar grey matter region (see ***Tau PET Region-of-Interest Definition)*** for explanation***.*** AD: Alzheimer´s disease (n= 13); Aβ- CU: Aβ-negative cognitively unimpaired individuals (n=13); bvFTD: behavioral variant of frontotemporal dementia (n =21); bvFTD ALS: bvFTD and amyotrophic lateral sclerosis (n=1), *C9orf72*: chromosome 9 open reading frame 72 (n = 11); *GRN*: progranulin (n = 1); *MAPT*: microtubule-associated protein tau (n= 1).

**Supplementary Fig. 2 Results of the PET voxel-based analyses and voxel-based morphometry** PET results are displayed using standardized uptake value ratios (Tau PET SUVRs) and W-scores (Tau PET W-scores), and the voxel-based morphometry using W-scores (Atrophy W-scores). AD: Alzheimer’s disease (n= 13); bvFTD: behavioral variant of frontotemporal dementia (n =21); bvFTD ALS: bvFTD and amyotrophic lateral sclerosis (n=1), *C9orf72*: chromosome 9 open reading frame 72 (n = 11); *GRN*: progranulin (n = 1); *MAPT*: microtubule-associated protein tau (n= 1). Please note that Tau PET W-score scale bars differ for AD and *MAPT*. The MAPT case scale bar is chosen because of the AD-like retention level seen.

**Supplementary Fig. 3 Co-localization of ^3^H-RO948 and AT8 in R406W FTD**

[^3^H]-RO948 autoradiography (30nM) and immunostaining with AT8 tau antibody performed on the same sections of a tissue sample from the inferior temporal gyrus of a case of frontotemporal dementia due to R406W *MAPT* mutation.

**Supplementary Fig. 4 Brain sections from two individuals with C9orf72 bvFTD who underwent [^18^F]RO948 PET and postmortem examination** A and B are from the parietal cortex of Case 1, A with H&E stain at x20 and B with pTDP-34 immunostaining at x200 magnification. C and D are sections from the frontal cortex of Case 2 at x20 (C) and x200 (D) magnification, immunostaining with pTDP-43 antibody.

**Supplementary Fig. 5 Results of the PET voxel-based analyses and voxel-based morphometry of the two individuals with *C9orf72* bvFTD who underwent [^18^F]RO948 PET and postmortem examination.** PET results are displayed using standardized uptake value ratios (Tau PET SUVRs) and W-scores (Tau PET W-scores), and the voxel-based morphometry using W-scores (Atrophy W-scores). Please note that W-score scale bars differ for AD dementia and *MAPT*.

**Supplementary References**

1. Zhang W, Arteaga J, Cashion DK, et al. A highly selective and specific PET tracer for imaging of tau pathologies. J Alzheimers Dis. 2012;31(3):601-12. doi: 10.3233/JAD-2012-120712.
2. Braak H, Braak E. Neuropathological stageing of Alzheimer-related changes. Acta Neuropathol. 1991;82(4):239-59. doi: 10.1007/BF00308809.
3. Mackenzie IRA, Neumann M, Baborie A, et al. A harmonized classification system for FTLD-TDP pathology. Acta Neuropathol. 2011;122:111–113. https://doi.org/10.1007/s00401-011-0845-8
